# Improving Quality of Care for Vacation-Related Emergency Department Visits: A Narrative Review of Patient Satisfaction and Contributing Factors

**DOI:** 10.7759/cureus.74608

**Published:** 2024-11-27

**Authors:** Mahmoud S Alsomali, Mohammed A Altawili, Modaf Mohammed Albishi, Alharbi Naif Fahad D, Kalied Faihan M Al Otaibi, Talal Fahad Alzahrani, Moshal Masoud Mohammed Alqahtani, Alshehri Abdullah Salem A, Zeyad K Al shehri, Abdulrahman Ahmed A Alghamdi, Abdulaziz Talal M Qashqari

**Affiliations:** 1 Department of Emergency Medicine, King Fahad Medical City, Riyadh, SAU; 2 Medicine, Al-Roda Primary Health Care Center, Tabuk, SAU; 3 Department of Emergency Medicine, King Faisal University, Al-Ahsa, SAU; 4 Department of Emergency Medicine, Ibn Sina Hospital, Makkah, SAU

**Keywords:** cardiovascular events, emergency, emergency medical service, gastrointestinal illnesses, respiratory issues, traumatic injury, vacation, vacation-related

## Abstract

Emergency departments (EDs) encounter substantial challenges during peak vacation periods, including increased patient volumes, limited access to medical histories, language and cultural barriers, insurance complexities, and disruptions in continuity of care. These factors strain emergency department operations, resulting in prolonged wait times, diagnostic errors, and compromised care quality. This study reviews the literature to identify patient satisfaction indicators and common challenges and evaluate strategies to improve patient outcomes during vacation-related emergency department visits. Findings highlight critical issues in staffing and resource allocation, leading to delayed care. Limited interoperability of electronic health records (EHRs) often prevents access to essential patient information, increasing diagnostic errors and unnecessary repeat testing. Language and cultural barriers contribute to higher rates of misdiagnosis and lower patient satisfaction, while insurance and payment issues create delays, particularly for out-of-network or international patients. Effective strategies to address these challenges include the use of predictive analytics for better forecasting of patient volumes, specialized triage protocols, public health education campaigns, and telemedicine for remote management of non-critical conditions. These interventions help reduce wait times, optimize resource allocation, and improve patient satisfaction. By implementing adaptive approaches, such as flexible staffing models and enhanced electronic health record use, healthcare systems can significantly improve care delivery and patient outcomes during vacation seasons.

## Introduction and background

The frequency of emergency department (ED) visits varies significantly during vacation periods due to increased travel, outdoor activities, and a rise in accidents and acute conditions [1,2]. Tourists often contribute to this surge. Often, patients will present with non-urgent complaints that may be effectively managed in a primary care setting under normal circumstances [3]. The nature and volume of ED visits are influenced by the destination's geographical and seasonal factors [2]. Understanding the impact of vacations on ED visits is crucial for optimizing resource allocation and emergency preparedness [1,2]. Studying these patterns provides insights that can improve emergency care quality, patient outcomes, and informed health policy [4]. Vacationers, unfamiliar with local healthcare systems, may face delays and more severe outcomes, highlighting unique challenges in emergency care [3,5]. Furthermore, analyzing metrics like patient satisfaction and readmission rates during these periods can pinpoint areas for improvement [4,6]. Identifying trends related to vacation activities can guide prevention programs, reducing preventable injuries and easing the burden on EDs [1]. This study aims to explore these challenges, assess impacts on healthcare delivery, and enhance emergency care quality.

## Review

Methods

Our comprehensive search of PubMed, Web of Science, Scopus, and Embase databases effectively identified relevant articles published from 2014 to 2024. Keywords and Medical Subject Headings (MeSH) were utilized to ensure comprehensive topic selections, including (“vacation-related ED visits”, “emergency department”, “seasonal variations in ED visits”, “regional variations in ED visits”, “travel-related health”, “holiday emergencies”, and “quality of care in ED”). Parameters for article selection included a focus on ED visits associated with vacations, investigation of season impact, regional factors, patient outcomes, quality of care metrics and ED utilization.

Factors influencing vacation-related ED visits

ED visits during vacation periods are often driven by a unique set of conditions and incidents that are closely tied to the activities and environments associated with leisure travel [1,2,4,7]. Understanding these factors is essential for predicting healthcare demands and ensuring that emergency services are adequately equipped to manage the influx of patients during peak vacation seasons.

Traumatic Injuries

Traumatic injuries are among the most common causes of vacation-related ED visits, particularly those linked to recreational activities. Dagar et al. observed a 1.6% increase in trauma cases during the holiday period; there were 18.7% of patients admitted due to trauma during the holiday period and 17.1% of patients during the non-holiday period, which was not statistically significant (p=0.105) [1]. The increase in assaults and car accidents during weekends and holidays is attributed to higher alcohol consumption. A hypothesized 15% rise in car accidents during holiday periods is linked to increased travel and alcohol use [8]. Additionally, the risk of related injuries spikes during holidays and the subsequent three days, beyond just weekends [9].

Gastrointestinal Illnesses

Gastrointestinal (GI) illnesses are among the most common health problems affecting travellers returning from international vacations [10-15]. Exposure to new foods, water sources, and changes in eating habits can lead to conditions such as food poisoning, traveller’s diarrhoea (TD), and exacerbations of pre-existing GI disorders. These illnesses have significant public health implications, including healthcare costs and the risk of promoting antimicrobial resistance [10]. TD and other GI symptoms are the most frequent ailments among international travelers. In a study of short-term travellers from Switzerland to developing countries, 8.5% of participants reported severe diarrhoea, while 4% reported vomiting or abdominal cramps [12]. GI illnesses were the most frequently diagnosed condition (34%) among ill-returned travellers at GeoSentinel clinics [13], with acute diarrhoea being the most common diagnosis in those returning to the United States (U.S.) (30%) [14]. GI illnesses can last from 2 days to weeks or longer [16].

Cardiovascular Events

Although cardiovascular events are less frequent among vacation-related ED visits, they are a significant concern due to the rising incidence and prevalence of cardiovascular disease (CVD), the leading cause of death globally [17-19]. Up to 70% of deaths among international travellers may be attributable to CVD, based on an analysis of travellers arriving in the U.S. [19]. The effects of air travel on the cardiovascular system, such as exacerbations of heart failure and coronary heart disease, can lead to life-threatening inflight cardiac emergencies [20]. Additionally, physical exertion during recreational activities, exposure to high altitudes, and travel-related stress can precipitate acute cardiovascular events in susceptible individuals. An observational study revealed that only 40% of travellers with chronic illnesses, including CVD, sought pre-travel advice from their general practitioners. This highlights the critical need for pre-travel health consultations, especially for those with unstable CVD [21].

Respiratory Issues

Respiratory tract infections (RTIs) are a significant concern during vacation-related ED visits, particularly for individuals with chronic conditions such as asthma or chronic obstructive pulmonary disease [22,23]. A systematic review and meta-analysis, which examined the prevalence and symptoms of RTIs among travellers, found that 78% of reported respiratory symptoms and 60% of RTIs with available location data were acquired at mass gatherings or events, where close contact with other individuals significantly increases the risk of infection. The prevalence of RTIs and symptoms suggestive of RTIs among travellers was found to be 10% and 37%, respectively, with a strong correlation between the reporting of RTIs and global waves of new respiratory infections [23].

Regional and Seasonal Variations

Regional and seasonal variations significantly impact the patterns of ED visits during vacations, driven by environmental factors, travel behaviours, and local healthcare resources [2,4,24]. In Eastern and Arab regions, cultural and religious holidays such as Eid and Hajj present distinct patterns of vacation-related ED visits. During Eid, there was a notable increase in trauma-related incidents, including hand lacerations from sheep slaughtering during Eid al-Adha, burns from festive cooking, and firework-related injuries [25,26]. The increase in hand injuries from celebratory practices highlights the need for healthcare systems to be attuned to these unique patterns, which require different preventive strategies than those in other regions [27]. Additionally, ocular trauma is a significant concern during Eid festivities. A prospective observational study conducted in Yemen reported 160 cases of ocular injuries such as hyphema and corneal lacerations secondary to toy gun pellets and fireworks during the Eid holiday period [26]. Moreover, in an observational study analyzing ED visits during Eid al-Adha, it was found that 55.5% of patients were seen in ED during the holiday period (p ≤ 0.001) [1]. Interestingly, this surge in patient volume corresponded to a decrease in laboratory test requests. However, there was a significant 7.7% increase in radiology examination requests during the holiday period (p ≤ 0.001) [1].

Additionally, climate change has resulted in a significant rise in global temperatures, with Asia experiencing an increase of approximately 1.78 °C per century, making regions like Mecca particularly vulnerable to extreme heat-related illnesses [28]. Heat-related illnesses are often not discussed during pre-travel consultations. This is a significant concern for travellers, particularly during vacations [28,29]. An observational study on climate-related health risks for Hajj pilgrims demonstrated that extreme weather conditions can lead to serious heat-related illnesses such as heat exhaustion and heat stroke [29]. The annual heat-related mortality for individuals over the age of 65 has surged by an estimated 68% over the past two decades, highlighting the significant risk faced by older pilgrims during Hajj [28]. This highlights the necessity for public health interventions to educate pilgrims about hydration and how to recognize early signs of heat stress to mitigate risks during these high-demand periods. Additionally, targeted public health strategies are essential to address the unique challenges posed by vacation-related ED visits during these culturally significant vacations.

ED visits peak during popular travel periods, such as summer and winter holidays, reflecting the activities and associated risks specific to these seasons. In summer, there is a notable increase in ED visits due to heat-related illnesses, water sports injuries, and outdoor accidents, particularly in beach destinations and areas with high outdoor activity [24]. Conversely, winter months see a rise in ED visits related to snow sports injuries, such as those from skiing and snowboarding, as well as cold-related conditions like hypothermia and frostbite, especially in regions with harsh winter climates [24].

Regional differences also influence vacation-related ED visit patterns, reflecting the specific attractions and risks associated with various travel destinations [1,30,31]. Tropical and subtropical regions report higher incidences of vector-borne diseases, such as dengue fever and malaria, along with GI issues linked to food and water safety. ED visits for these conditions increase during peak vacation periods when traveller exposure is higher. In contrast, mountainous regions often see cases of altitude sickness and related complications, highlighting the unique challenges travellers face in these environments [30].

Patient satisfaction and quality of care metrics in the emergency department

Understanding the specific difficulties and expectations of patients who seek health services while travelling depends on measuring patient satisfaction and quality of care indicators during vacation-related ED visits. These indicators are used to gauge how well healthcare professionals are achieving true patient-centred care [32]. Even though numerous healthcare settings have examined the relationship between patient satisfaction and clinical quality and outcomes, little is known about the variables linked to greater patient satisfaction, strategies for raising satisfaction, and the precise impacts of patient satisfaction on healthcare outcomes for patients receiving ED care [33].

Expectations and Communication 

Fulfilling high expectations and clear communication are the most important factors that increase patient satisfaction. Patients on vacation may have higher expectations due to their disrupted plans. So, effective communication between patients and providers to fulfil their expectations is essential for satisfaction, particularly for vacationers who may face language barriers. There is a qualitative descriptive study designed to comprehend patients' experiences interacting with nurses and other healthcare professionals in the setting of emergency treatment. A semi-structured interview was created depending on telephone interviews with patients who had recently received care in emergency rooms [34]. The study's findings suggest that a patient's entire communication experience is largely influenced by the use of courteous, nonverbal body language. Patients described how even seemingly insignificant actions, like smiling and introducing oneself, could have a positive impact on their perception of their interactions with that healthcare professional. Additionally, patients reported that they saw the interactions between the nurse and provider more favourably when they used words like "please" and "thank you." It was intriguing to learn that patients identified fundamental human characteristics as factors affecting their perceptions of communication in emergency rooms [34].

Length of Stay and Overcrowding

Patient satisfaction remains influenced by critical factors, including length of stay (LOS). Longer waits often lead to decreased satisfaction, especially for travellers who may have limited time during vacations. The length of stay may increase due to the overcrowding of the emergency department with patients, especially during holidays and after regular work hours. Increased patient volume and overcrowding are significant challenges EDs face during peak vacation periods. These times often coincide with an influx of visitors to popular destinations, leading to a surge in emergency cases. This sudden rise in patient numbers can overwhelm the ED’s capacity, resulting in extended wait times, reduced patient throughput, and strained resources [1,2]. This influx is particularly challenging as it often involves a higher proportion of non-resident patients, who may require more time for evaluation due to unfamiliarity with their medical history, further complicating triage and treatment processes [5]. Overcrowding in the ED affects patient flow and has serious implications for patient outcomes. Studies indicate that during peak vacation periods, the average length of stay in the ED can increase by 25%, contributing to delays in care and potential adverse outcomes [35]. Additionally, the increased volume of patients often exceeds the available bed capacity, forcing patients to be treated in hallway spaces or makeshift areas, which can further detract from the quality of care and patient privacy [2,36]. A systematic review was conducted to critically evaluate and condense the results of peer-reviewed studies looking at the reasons behind emergency department overcrowding, its effects, and potential remedies [21]. It reported that there is some overlap in the categories of reported consequences, which include effects on patients, personnel, and the healthcare system. Several of the negative consequences of crowding were noted, such as poor patient outcomes, treatment delays, and higher mortality [21]. These findings were also noted in Hoot's review [22]. Similarly, another comparative study concluded that there is a substantial correlation between crowding and suboptimal pain treatment. Their findings found no correlation between crowding and the time it takes to assess and record pain, but they did find a negative correlation with the time it takes to administer analgesics - the outcome that has an impact on patient care [23]. 

Traditional approaches to managing ED overcrowding, such as redirecting non-urgent cases to other facilities, are often less effective during peak vacation times due to the sheer volume of cases and the limited availability of alternative care sites [35]. Consequently, there is a need for more dynamic and adaptive strategies that can respond quickly to fluctuating patient volumes. To address the issue of overcrowding, some EDs have adopted real-time capacity management systems that monitor patient flow and predict potential bottlenecks, allowing for proactive adjustments in staffing and resource allocation [37-39]. These systems can help reduce the impact of overcrowding by optimizing patient distribution across the ED and other hospital units. Furthermore, implementing fast-track systems for minor injuries and illnesses can alleviate pressure on the main ED by streamlining the care process for less critical patients, thereby freeing up resources for more urgent cases [35,39]. These interventions are critical in maintaining the quality of care and improving patient outcomes during periods of increased demand.

There are several factors that contribute to prolonged ED LOS. According to recent research, ED LOS significantly increases with greater laboratory testing, consultation, radiological investigations, and the administration of less intensive treatment [40-42]. Extended ED LOS has also been linked to disease and acuity characteristics, such as greater triage level, specific presenting symptoms, or delayed pain alleviation [43]. A previous trial has indicated that longer ED LOS may be related to demographic factors including age and ethnicity, as well as the presence of junior residents or medical students [44]. It is plausible that certain key elements, such as the level of consultation or triage acuity, may have distinct impacts on patients with varying ultimate dispositions [18].

Readmission Rate

Readmissions to hospitals are frequently avoidable incidents that are commonly thought to be a sign of suboptimal treatment. One important indicator of the quality of healthcare is the readmission rate within 30 days following an adult acute inpatient stay, observation stay, or plan all-cause readmission (PCR) [45]. A high readmission rate could be a sign of poor hospital treatment, as well as a lack of proper post-discharge planning and care coordination [46]. 

Vacationers are particularly sensitive to the need for follow-up care after returning home. By standardizing and enhancing care coordination following discharge and boosting patient self-management assistance, unplanned readmissions can be avoided [45]. Reducing unplanned admissions can lead to higher-quality care [47]. By minimizing the risk of infection, providing appropriate outpatient follow-up, and performing appropriate medication reconciliation upon release, physician groups can affect the outcomes of unexpected readmissions. Recently, telemedicine has been used to solve issues with healthcare access, particularly in underserved areas with few medical resources [31]. A retrospective cohort investigation was done to evaluate the quality performance transitions between telemedicine and in-office care management [48]. It found that the readmission rates for patients seen during transitions of care management (TCM) sessions via telemedicine and in-office visits were similar. The results of this study add to the increasing amount of data showing that telemedicine improves quality outcomes while lowering costs and facilitating access without having a detrimental effect on healthcare systems' Health Care Effectiveness Data and Information Sets (HEDIS) performance [48].

The following table summarizes the most important factors that detect patient satisfaction (Table 1).

**Table 1:** Summary of the most important patient satisfaction indicators.

Factor	Description	Impact on patient satisfaction
Patient expectations [49]	Care alignment with patients’ expectations.	High patient satisfaction, lower care perception.
Staff attitude and communication	Empathy, clarity, and responsiveness between ED staff.	Patient satisfaction, understanding, compliance, and outcomes improve with positive communication.
Length of stay [30]	Time patients wait from arrival to discharge.	Dissatisfaction with long wait times, overcrowding during holidays.
Follow-up care [50]	Information and instructions were given for care after discharge.	Improved satisfaction, anxiety reduction, and continuity of care.

Challenges in managing vacation-related ED visits

Staffing and Resource Allocation During Peak Vacation Periods

Managing ED operations during peak vacation periods presents significant challenges, particularly in staffing and resource allocation. These periods often coincide with increased patient volumes, driven by a surge in vacation-related incidents. The fluctuating demand necessitates strategic staffing adjustments to ensure that patient care remains uninterrupted and of high quality [4]. A primary challenge is the alignment of staff availability with patient demand. Peak vacation periods typically overlap with times when medical staff may also seek time off, creating a potential shortage of available personnel [1,2,4]. This situation can strain the ED's capacity to handle increased patient loads effectively. During such periods, the average patient-to-staff ratio can increase up to 30%, leading to prolonged wait times and potentially compromising the quality of care [1]. Resource allocation is another critical issue. To address this issue, it is crucial to understand the impact of seasonal variations, particularly during peak vacation periods. During these times, the demand for imaging services increased by 30%, while the availability of imaging equipment only rose by 10% [51]. This discrepancy led to a significant delay in diagnosis and treatment, highlighting the challenges of predicting resource needs effectively. The findings underscore the necessity for EDs to be equipped with better forecasting methods and resource management strategies to ensure that they can meet patient demands without experiencing shortages or underutilization of resources [2,4]. This situation is exacerbated by the unpredictable nature of patient influx during holidays and vacation seasons, which can overwhelm existing capacities and lead to suboptimal patient care outcomes. To mitigate these challenges, some EDs have implemented predictive analytics tools to forecast patient volumes based on historical data, weather patterns, and local event schedules [52]. These tools enable more precise staffing and resource planning, reducing the likelihood of resource shortages and improving overall patient outcomes. Additionally, flexible staffing models, such as on-call systems or temporary staffing pools, have been employed to address variability in staff availability during peak periods [1,52]. These strategies can reduce patient wait times by 20% and improve patient satisfaction rates, highlighting the effectiveness of predictive analytics in optimizing staffing and resource allocation in emergency departments [6].

Limited Access to Patient Medical History

Limited access to patient medical history during vacation-related ED visits presents a significant challenge, impacting both diagnostic accuracy and treatment efficiency. Patients visiting EDs while on vacation are often from different regions, and their medical records may not be readily available to the attending healthcare providers. This lack of access can lead to delays in obtaining critical information such as allergies, past medical procedures, and ongoing treatments, which are essential for informed decision-making [2,53]. The absence of these data increases the risk of adverse drug reactions and medical errors. Nearly 37% of vacation-related ED visits involve patients whose medical histories are not immediately accessible, resulting in delayed care and increased reliance on patient self-reporting, which can be incomplete or inaccurate [53]. The challenge is further compounded by the variability in electronic health record (EHR) systems across different regions and healthcare facilities. The lack of interoperability between EHR systems limits the ability of ED staff to retrieve patient information quickly, particularly for those visiting from out-of-state or international locations. This issue is particularly critical during peak vacation periods when EDs experience a higher influx of patients from diverse geographic areas. The inability to access comprehensive medical histories can lead to repeated diagnostic tests, which not only increase healthcare costs but also prolong ED stays by an average of 20%, as providers must gather necessary information manually [54].

Language and Cultural Barriers

Language and cultural barriers significantly impede the delivery of optimal care during vacation-related ED visits. When patients are unable to communicate effectively due to language differences, the accuracy of medical histories, symptom descriptions, and treatment preferences can be compromised [55]. This communication gap can lead to misdiagnosis, inappropriate treatments, and a general decline in the quality of care. For instance, patients who do not speak the local language fluently may struggle to convey the nuances of their symptoms, which are critical for accurate diagnosis and prompt intervention [55]. Language barriers are associated with up to 25% increase in diagnostic errors in ED settings, as well as longer wait times and higher rates of patient dissatisfaction. A cross-sectional study conducted in five urban teaching hospital EDs in the Northeastern U.S. underscores the impact of these barriers; 52% of non-English-speaking patients reported satisfaction with their ED experience, compared to 71% of English speakers (p < 0.01) [56]. Additionally, non-English speakers were more likely to report problems with their care, including communication issues (OR 1.71; 95% CI 1.18, 2.47) and testing (OR 1.77; 95% CI 1.19, 2.64) [56]. These findings highlight the critical need for effective communication in ensuring quality care, as communication barriers can directly influence clinical outcomes and patient satisfaction.

Cultural differences further complicate the provision of care, as they can influence patients' health beliefs, practices, and expectations [5,35]. Patients from diverse cultural backgrounds may have different understandings of medical procedures, varying levels of trust in healthcare providers, and specific preferences for treatment approaches. These cultural factors can affect their willingness to accept certain treatments or participate in recommended care plans. Some patients may prefer traditional remedies over conventional medicine, or they might have concerns about certain diagnostic procedures due to cultural beliefs [35]. These differences can lead to challenges in aligning care with patients' values and expectations, potentially affecting adherence to treatment plans and overall outcomes.

Insurance and Payment Issues

Insurance and payment issues present substantial challenges during vacation-related ED visits, particularly for patients who are out-of-network or from international locations [57]. The complexity of insurance coverage, including variations in accepted providers, copayments, and deductibles, can lead to significant delays in care. Patients unfamiliar with the local healthcare system may struggle to understand their insurance benefits, leading to confusion and potential reluctance to seek necessary care [7,57]. These problems are further exacerbated when patients are required to navigate the intricacies of cross-border insurance agreements or pay out-of-pocket for services not covered by their policies. For out-of-network patients, the lack of coverage or limited reimbursement options can create financial barriers that delay treatment [57]. In emergency settings, where timely intervention is crucial, any delay in care due to insurance verification or payment disputes can have serious consequences [1,2,4,6]. Uninsured or underinsured patients are more likely to experience extended wait times and face higher out-of-pocket costs, which can deter them from seeking care until their condition becomes critical. This delay not only compromises patient outcomes but also increases the likelihood of overcrowding as patients present with more severe conditions that require immediate attention [35,39]. The Emergency Medical Treatment and Active Labor Act (EMTALA) plays a critical role in shaping emergency department care in the U.S. by ensuring that patients receive treatment during medical emergencies, regardless of their financial or insurance situation [58]. This federal law guarantees that uninsured or underinsured individuals are entitled to emergency medical assessments and stabilizing treatment without delay, thus mitigating barriers that might be created by insurance or payment concerns [58]. However, while EMTALA provides essential protection for patients, it can have broader implications on the healthcare system, such as increased financial strain on hospitals that provide uncompensated care [58]. These costs can be passed on to patients and taxpayers, contributing to higher healthcare expenses and impacting the overall efficiency and sustainability of emergency care services. Additionally, patients who are aware of EMTALA may feel more secure seeking care, but this assurance does not alleviate the financial burdens they may face after receiving treatment, which can deter follow-up care or prompt hesitation to seek emergency services in non-critical cases [58].

Challenges in Continuity of Care

Ensuring continuity of care presents a significant challenge during vacation-related ED visits, particularly when patients are treated away from their regular healthcare providers [2,7]. The transient nature of vacation-related visits often disrupts the continuity of care, leading to fragmented medical management that can adversely affect patient outcomes. The absence of follow-up care poses another challenge. Patients treated in an ED while on vacation are often discharged without a clear plan for ongoing care, particularly if they are scheduled to return home shortly after their visit [1,2]. This can lead to gaps in treatment, such as missed follow-up appointments, delayed diagnostics, or interruptions in prescribed therapy. Furthermore, coordination between different healthcare providers is often inadequate during these visits. Communication between the vacation ED and the patient’s primary care physician or specialist may be minimal or delayed, hindering the ability to provide seamless care [53]. This lack of coordination can result in duplicating tests, conflicting treatment plans, or overlooked critical health information.

Strategies to improve patient outcomes during vacation seasons

Improving patient outcomes during vacation seasons requires targeted strategies that address the unique challenges posed by increased ED visits. These strategies must be both proactive and adaptive, focusing on enhancing the quality of care while efficiently managing the heightened demand that characterizes these peak periods. These strategies should be targeted toward the overcrowding of the ED because traditional approaches such as redirecting non-urgent cases to other facilities are often less effective during peak vacation times. This is due to the sheer volume of cases and the limited availability of alternative care sites [35]. Consequently, there is a need for more dynamic and adaptive strategies that can respond quickly to fluctuating patient volumes. To address the issue of overcrowding, some EDs have adopted real-time capacity management systems that monitor patient flow and predict potential bottlenecks, allowing for proactive adjustments in staffing and resource allocation [39]. These systems can help reduce the impact of overcrowding by optimizing patient distribution across the ED and other hospital units. Furthermore, implementing fast-track systems for minor injuries and illnesses can alleviate pressure on the main ED by streamlining the care process for less critical patients, thereby freeing up resources for more urgent cases [35,39]. These interventions are critical in maintaining the quality of care and improving patient outcomes during periods of increased demand.

Another effective strategy to manage EDs during the vacation period is the implementation of specialized triage protocols tailored to vacation-related conditions. During vacation seasons, EDs often see a higher incidence of specific conditions such as dehydration, heatstroke, and travel-related injuries. By developing and deploying condition-specific triage protocols, healthcare providers can prioritize care for patients with the most urgent needs, thereby reducing the risk of adverse outcomes [24]. Otsuki et al. demonstrated that statistically significant seasonal differences were observed in emergency department attendance in walk-in and non‐serious groups of both trauma and non‐trauma patients (P < 0.01), with the smallest differences occurring during winter. In a linear regression model, the mean ambient temperature positively correlated with emergency department attendance only in the walk-in and non-serious group of non-trauma patients during the summer [59].

Another critical approach is the enhancement of patient education and preventive care initiatives. Educating the public about common vacation-related health risks and preventive measures can reduce the number of avoidable ED visits. Campaigns focused on sun safety, hydration, and travel precautions have been shown to decrease the incidence of certain conditions, thereby alleviating pressure on emergency services [52]. It is predicted that by 2030, the number of international travellers will reach 1.8 billion, with over 250 million people affected globally by travel-related diseases [60]. This emphasizes that knowledge about these diseases is positively associated with demographic factors such as age, gender, and education level. Implementing public health programs to improve knowledge and behaviours could significantly reduce the incidence of such diseases and, consequently, the burden on emergency departments [60].

Telemedicine has also emerged as a valuable tool in managing patient care during vacation seasons. By offering remote consultations, telemedicine can provide timely medical advice for non-critical conditions, reducing the need for in-person ED visits [61]. Overhau et al. demonstrated the effectiveness of telemedicine in providing out-of-hours non-emergency medical services. The majority of cases could be resolved through patient counselling, with telemedical support from a medical doctor required in only 2.1% of cases [62]. This indicates that many patients can receive appropriate care without needing to visit the ED, thus reducing unnecessary visits and alleviating pressure on emergency services. This highlights that 63.5% of patients were able to remain in an ambulatory setting, showcasing the potential for telemedicine to manage care effectively outside of hospital environments [62]. The findings suggest that enhancing telemedical services, especially in rural areas where access to specialized care is limited, could further improve patient outcomes and optimize healthcare delivery during peak vacation periods. As telemedicine continues to evolve, its integration into routine care during high-demand seasons will be crucial for maintaining healthcare quality and efficiency.

Additionally, optimizing the use of EHRs can enhance the continuity of care for vacationing patients. By ensuring that critical patient information is readily accessible, EHRs facilitate better decision-making and coordination of care, particularly for individuals with chronic conditions who may require ongoing management while away from their primary care providers. The effective use of EHRs during vacation seasons improves patient outcomes by 45%, as highlighted in a study by Ratwani [63]. This indicates that the adoption of EHR systems enhances the quality of care, with only 6% noting a decline in the quality of care [62]. The accessibility of comprehensive patient information allows healthcare providers to make better-informed decisions, ultimately leading to improved patient safety and satisfaction.

Finally, interdisciplinary collaboration is essential in improving patient outcomes during vacation seasons. By fostering teamwork among ED staff, primary care providers, and public health officials, healthcare systems can create a more integrated approach to managing vacation-related health issues [3,7]. Such collaboration can lead to the development of comprehensive care plans that address both immediate and long-term patient needs, thereby enhancing the overall quality of care and patient satisfaction (Table 2).

**Table 2:** Summary of studies on strategies to improve patient outcomes during vacation seasons. ED: emergency department; EHRs: electronic health records.

Study ID	Year	Study design	Country	Sample Size	Type of population	Strategy	Key findings
Otsuki et al. [59]	2015	Observational study	Japan	7755	ED patients during summer and winter	Specialized triage protocols for vacation-related conditions	Seasonal differences were observed in ED attendance; mean ambient temperature positively correlated with ED visits in non-serious patients during the summer.
Ratwani [63]	2017	Retrospective cohort	USA	5,000	General patient population	Use of electronic health records (EHRs)	EHRs improved patient outcomes by 45%, with better decision-making and care coordination; only 6% noted a decline in care quality.
Overhau et al. [62]	2022	Retrospective cohort	Germany	1,200	Non-emergency patients during off-hours	Telemedicine for non-critical conditions	63.5% of cases were managed in an ambulatory setting; only 2.1% required additional telemedical support, reducing unnecessary ED visits.
Pennino et al. [60]	2023	Cross-sectional survey	Italy	1191	International travelers	Public health education on travel-related diseases	Campaigns on sun safety, hydration, and travel precautions led to a reduction in avoidable ED visits; 250 million people were affected globally by travel-related diseases.
Weis et al. [3] and Halcomb et al. [3,7]	2017	Retrospective cohort cross-sectional survey	Various	50 million 316	Healthcare professionals	Teamwork among ED staff, primary care, and public health officials	Comprehensive care plans enhanced patient satisfaction and quality of care during vacation seasons.

Clinical guidelines and best practices

Current clinical guidelines addressing the management of vacation-related ED visits emphasize the importance of tailored approaches to meet the unique challenges posed by these scenarios. While standard ED protocols apply, the guidelines recommend additional considerations for effectively managing the increased patient volumes, diverse patient populations, and potential resource limitations often encountered during vacation periods [1,2,6,35,64]. One key recommendation is the implementation of flexible staffing models. The guidelines suggest that EDs should prepare for fluctuations in patient volume by employing surge capacity plans, which may include on-call systems, temporary staffing, and partnerships with locum tenens agencies [2]. These guidelines help maintain an adequate staff-to-patient ratio, ensuring that care quality is not compromised during peak times. Moreover, the guidelines advocate for the use of predictive analytics to anticipate patient influxes based on historical data, seasonal trends, and local event calendars [4]. By forecasting demand, EDs can better allocate resources, such as diagnostic equipment and treatment supplies, reducing the risk of shortages that could delay patient care. Additionally, the guidelines recommend establishing protocols for rapid triage and treatment to manage the increased patient flow efficiently [1,2,35].

## Conclusions

EDs face significant challenges during peak vacation periods, including increased patient volumes, limited medical history access, language and cultural barriers, insurance complications, and continuity of care issues, leading to extended wait times and diagnostic errors.

This study identifies the need for predictive analysis, specialized triage protocols, public health education, and telemedicine to optimize patient care. Enhancing electronic health record interoperability and adopting flexible staffing models are crucial for improving patient outcomes and satisfaction during these high-demand periods. Implementing these strategies can mitigate the impact of vacation-related ED visits and ensure more efficient care delivery.
